# Direct medical charges of pediatric traumatic brain injury in multiple clinical settings

**DOI:** 10.1186/2197-1714-1-13

**Published:** 2014-05-06

**Authors:** Christy L Collins, Keith Owen Yeates, Thomas L Pommering, Rebecca Andridge, Victor G Coronado, Julie Gilchrist, R Dawn Comstock

**Affiliations:** 1 Center for Injury Research and Policy, Nationwide Children’s Hospital, Columbus, OH USA; 2 Department of Pediatrics, College of Medicine, The Ohio State University, Columbus, OH USA; 3 Center for Biobehavioral Health, Nationwide Children’s Hospital, Columbus, OH USA; 4 Department of Pediatrics and Family Medicine, College of Medicine, The Ohio State University, Columbus, OH USA; 5 Division of Sports Medicine Nationwide Children’s Hospital, Columbus, OH USA; 6 Division of Biostatistics, College of Public Health, The Ohio State University, Columbus, OH USA; 7 Division of Unintentional Injury Prevention, National Center for Injury Prevention and Control, Centers for Disease Control and Prevention, Atlanta, GA USA; 8 Colorado School of Public Health, Epidemiology and Pediatric Injury Prevention, Education, and Research (PIPER) Program, Aurora, CO USA

**Keywords:** Traumatic brain injury, Children, Medical charges

## Abstract

**Background:**

Data limited to emergency department and inpatient visits undoubtedly underestimate the medical charges associated with traumatic brain injury. The objective of this study was to estimate the direct medical charges of pediatric traumatic brain injuries across all clinical settings in one large, pediatric hospital in the United States.

**Methods:**

Traumatic brain injuries sustained by children ≤20 years of age treated across all clinical settings at one large pediatric hospital from August 1, 2010-July 31, 2011 were identified using ICD-9-CM codes 310.2, 800–801.9, 803–804.9, 850–854.16, and 959.01.

**Results:**

3,971 individuals ≤20 years of age were seen during 5,429 traumatic brain injury-related visits. Total medical charges for pediatric traumatic brain injury-related visits were $13,131,547. Inpatient (68.7%) and emergency department (16.1%) visits accounted for the highest proportion of TBI-related charges; however, >15% of all charges were associated with visits to clinic outpatient, urgent care, and diagnostic/therapy outpatient settings. Fracture of the vault or base of the skull (37.1%) and brain injury with contusion, laceration, or hemorrhage (27.1%) accounted for the largest proportion of total charges. Although unspecified head injuries made up almost half of all TBI-related visits (47.4%), they accounted for only 12.6% of total charges. Mild traumatic brain injuries accounted for 92.0% of all traumatic brain injury-related visits but only 44.7% of all traumatic brain injury-related charges. Mild traumatic brain injuries treated in the emergency department had a higher median total charge than those treated in urgent care (p < 0.0001) or clinic outpatient setting (p < 0.001).

**Conclusions:**

This study, the first to evaluate the direct medical charges of pediatric traumatic brain injury across all clinical settings at one large pediatric hospital, found that pediatric traumatic brain injuries present to a wide variety of clinical settings, and differences exist in total charges by diagnosis, severity of the injury, and clinical site/setting. Investigating traumatic brain injuries across the full spectrum of clinical care is needed for a better understanding of the true medical cost and public health burden of pediatric traumatic brain injury.

**Electronic supplementary material:**

The online version of this article (doi:10.1186/2197-1714-1-13) contains supplementary material, which is available to authorized users.

## Background

Traumatic brain injury (TBI) has received increasing attention over the past decade because of its incidence, economic cost, human impact, and preventability. Although the true incidence of TBI is unknown, in 2009 in the United States (US), there were at least 2.4 million emergency department (ED) visits, hospitalizations, and deaths related to a TBI, either alone or in combination with other injuries (Coronado et al. 2012a). As TBIs can have both short and long term effects including impairment of physical, cognitive, or emotional functions, (National Center for Injury Prevention and Control 2003; Cassidy et al. 2004) many TBI prevention recommendations have been made; yet, the reported incidence of TBI continues to increase (Faul et al. 2010; Thurman et al. 1999; National Center for Injury Prevention and Control 2003).

Estimated in 2009 US dollars, the total lifetime health care cost of fatal, hospitalized, and non-hospitalized TBI was approximately $221 billion. Of this, $57.8 billion were related to hospitalization-related costs (Orman et al. 2011). Mild TBIs (mTBI), which may account for 70%-90% of all TBIs, (Faul et al. 2010; Cassidy et al. 2004) often present to clinical settings other than the ED (e.g., urgent care clinics, sports medicine clinics, concussion clinics, etc.) (Schootman and Fuortes 2000; National Center for Injury Prevention and Control 2003; Cassidy et al. 2004; McCrea et al. 2004; Kirkwood et al. 2008; Thurman et al. 1999). Therefore, not only has the incidence of TBI been underestimated, but the resultant cost of TBI has certainly been underestimated as well.

Pediatric TBIs, including mTBIs, are of special concern. Children <14 years of age account for nearly half a million (473,947) TBI-related ED visits in the US annually, (Faul et al. 2010) and the highest rates of TBI-related ED visits occur among children <5 years and adolescents 15 to 19 years of age (Faul et al. 2010). Like adults, children who sustain TBIs are at risk of short term physical, cognitive, emotional, and social impairments (Moran et al. 2012; Taylor 2004; Yeates et al. 2012). Additionally, TBI may continue to negatively affect development and brain maturation as children grow into adulthood (Anderson et al. 2004) (Yeates et al. 2005; Schwartz et al. 2003).

Several studies have investigated the economic burden of pediatric TBI; however, the definition of “cost” has varied widely across these studies (Rockhill et al. 2010; Schneier et al. 2006; Shi et al. 2009; Jaffe et al. 1993; Brener et al. 2004). One study from 2006 found pediatric TBI-related hospitalization charges in the US totaled approximately $2.6 billion dollars (Shi et al. 2009). Again, this figure underestimates the true cost of pediatric TBI as data were utilized from only one clinical setting (i.e., inpatient data) (Schneier et al. 2006; Shi et al. 2009). Another study that included pediatric TBI-related direct and indirect costs from various clinical settings found, from 1997 through 2000, the cost of mild to moderate pediatric TBI-related services totaled $77.9 million annually in the US (Brener et al. 2004). However, the generalizability of previous studies that used cost data from multiple clinical settings may be limited as they included a relatively small sample of children (Brener et al. 2004; Jaffe et al. 1993). The objective of this study was to evaluate the true direct medical charges of pediatric traumatic brain injuries by analyzing total medical charges across multiple clinical settings at one large United States (US) Midwest Children’s Hospital.

## Methods

TBIs from all causes sustained by children ≤20 years of age treated at one large Midwest Children’s Hospital from August 1, 2010 through July 31, 2011 were identified using ICD-9-CM codes 310.2, 800–801.9, 803–804.9, 850–854.16, and 959.01 as the principal diagnosis. Previous studies have found that sports-related activities account for a large proportion of pediatric TBIs; (Leibson et al. 2011; Meehan and Mannix 2010) therefore, an academic year time period was used in an effort to capture sports-related initial injury visits as well as any related follow-up visits in our study period. We captured all TBIs seen across the wide array of clinical settings at this Children’s Hospital including inpatient, ED, urgent care, clinic outpatient, and diagnostic/therapy outpatient settings.

Variables of interest included sex, age of patient at first visit, race (white, Black/African American, two or more races, other, and unknown), zip code from patient’s home address, clinical site/setting (inpatient, ED, urgent care, clinic outpatient, diagnostic/therapy outpatient, observation, and organ procurement), principal diagnosis, primary payor (commercial, Medicaid, self-pay, and other), and total medical charges. Data from the US Census Bureau were used to obtain average median household income of the zip codes from the patients’ home address (patient zip code and/or median household income were missing for 12 individuals). Individual ICD-9-CM codes were grouped into 5 diagnosis categories: 1) concussion (ICD-9-CM codes 850.0-850.9 and 310.2), 2) head injury, unspecified (959.01), 3) brain injury with contusion, laceration, or hemorrhage (851.0-854.16), 4) fracture of the vault or base of the skull (800.0-801.9), and 5) other and unqualified multiple fractures of the skull (803.0-804.9). Based on criteria from past research, (Bazarian et al. 2006) the following ICD-9-CM codes meet the CDC criteria for a case of mild TBI:


Data for mTBI are not shown in all tables but, rather, are largely presented in the text. Total medical charges included all direct charges such as physician charges, pharmaceutical charges, laboratory charges, facility charges, etc.

If an individual had two or more TBI visits during the study period that were more than 90 days apart, two clinicians from the research team (KOY and TLP) independently reviewed the medical records to determine if the visits were related to the same injury event or if they represented multiple independent injury events. The two clinicians reviewed the medical records of 77 individuals with two or more TBI visits and agreed that for 30 individuals the visits were related to the same injury while for 43 individuals the visits represented multiple independent injury events. Medical records were reviewed a second time by both clinicians for the 4 (5.2%) individuals on which they initially disagreed, and an agreement was reached that 1 individual had two or more visits related to the same injury and 3 had two or more visits representing multiple independent injury events.

Data were analyzed using SPSS software, version 19.0. Because average total charge per patient was right-skewed, median total charge per patient was used. Differences in the median total charge per patient by gender were evaluated by a Mann Whitney U test. Differences in total charge per patient by race and primary payor were evaluated by a Kruskal-Wallis test. Linear regression was used to assess total charge per patient by age of the patient at the first visit, average median household income for the patient’s zip code, and number of visits per patient. This study was approved by the Institutional Review Board at Nationwide Children’s Hospital.

## Results

### TBI patients demographics and charges

From August 1, 2010 through July 31, 2011, 3,971 patients ≤20 years of age were seen for a TBI injury at one large Midwest Children’s Hospital. The mean age of patients was 7.77 years (SD: 5.88 years), and 61.9% were male (Table 1). The majority of patients were white (64.0%), 22.0% were Black/African American, 5.9% were other races, 4.4% were two or more races, and 3.6% were unknown. The primary payors for the majority of patients were commercial insurance (52.4%) and Medicaid (43.2%) followed by self-pay (3.2%) and other source (1.2%) (Table 1). The average median household income within the patients’ home zip codes was $56,242 (standard deviation (SD): $22,797).

**Table 1:** **Demographic characteristics of pediatric patients with a principal diagnosis of traumatic brain injury at one midwest children’s hospital from August 1, 2010 through July 31, 2011, overall and by number of visits per individual**

	Total # individuals (%)	# Individuals with one visit* (%)	# Individuals with two or more visits* (%)	
			Two or more related visits	Two or more unrelated visits
**Gender**				
Male	2,459 (61.9%)	1,944 (61.0%)	485 (65.5%)	30 (65.2%)
Female	1,512 (38.1%)	1,241 (39.0%)	255 (34.5%)	16 (34.8%)
**Total**	**3,971 (100.0%)**	**3,185 (100.0%)**	**740 (100.0%)**	**46 (100.0%)**
**Age, years**				
Mean (SD)	7.77 (5.88)	6.93 (5.66)	11.39 (5.41)	7.04 (6.12)
**Race**				
White	2,543 (64.0%)	1,961 (61.6%)	551 (74.5%)	30 (65.2%)
Black/African American	875 (22.0%)	757 (23.8%)	110 (14.9%)	8 (17.4%)
Other	236 (5.9%)	204 (6.4%)	29 (3.9%)	3 (6.5%)
Two or more races	175 (4.4%)	143 (4.5%)	27 (3.6%)	5 (10.9%)
Unknown	143 (3.6%)	120 (3.8%)	23 (3.1%)	0 (0.0%)
**Total**	3,971 (100.0%)*	3,185 (100.0%)*	**740 (100.0%)**	**46 (100.0%)**
**Primary Payor**				
Commercial	2,081 (52.4%)	1,552 (48.7%)	507 (68.5%)	22 (47.8%)
Medicaid	1,715 (43.2%)	1,487 (46.7%)	205 (27.7%)	23 (50.0%)
Self-pay	126 (3.2%)	105 (3.3%)	21 (2.8%)	0 (0.0%)
Other	49 (1.2%)	41 (1.3%)	7 (0.9%)	1 (2.2%)
**Total**	**3,971 (100.0%)**	**3,185 (100.0%)**	740 (100.0%)*	**46 (100.0%)**
**Average median household income by patient zip code, dollars**				
Mean (SD)	56,242 (22,797)	55,167 (22,519)	60,986 (23,350)	54,551 (23,759)
**Total charges per patient, dollars**	**Total charge per patient**	**Total charge per patient among individuals with one visit**	**Total charge per patient among individuals with two or more related visits**	**Total charge per patient among individuals with two or more unrelated visits**
Mean (SD)	3,307 (26,225)	1,832.55 (11,129.20)	9,757 (55,765)	1,629 (1,993)
Range	1,016,317	359,606	1,016,317	11,217
25^th^ percentile	186	167	701	484
50^th^ percentile	348	320	1,254	878
75^th^ percentile	1,057	728	2,987	2,617

* Totals do not sum to 100.0% due to rounding.

The median total charge per patient was $348 (Table 1). Overall, as age of the patient increased, total charge per patient also increased (p = 0.024). As expected, as number of visits increased, the total charge per patient increased (p < 0.001) (Table 2). There were also significant differences in median total charge per patient by gender (p = 0.033), race (p < 0.001), and primary payor (p = 0.001) (Table 2). There was no significant difference in average median household income of the patient’s zip code. Among patients with mTBIs, there were also significant differences in median total charge per patient by gender, race, and primary payor.

**Table 2:** **Median total charge per pediatric patient with a principal diagnosis of traumatic brain injury at one midwest children’s hospital from August 1, 2010 through July 31, 2011**

	Median total charge per patient
**Gender**	
Male	$375
Female	$332
**Race**	
White	$415
Black/African American	$321
Other	$327
Two or more races	$321
Unknown	$340
**Primary Payor**	
Commercial	$400
Medicaid	$326
Self-pay	$470
Other	$320
**Number of visits**	
1	$320
2	$864
3	$1,231
4	$1,808
5 or more	$3,531

### TBI visit characteristics

Total charges for all 5,429 TBI-related visits were $13,131,547 (Table 3). Although unspecified head injuries (ICD-9-CM code 959.01) made up almost half of all TBI-related visits (47.4%), they accounted for only 12.6% of total charges (Table 3). Fracture of the vault or base of the skull (37.1%) and brain injury with contusion, laceration, or hemorrhage (27.1%) accounted for the largest proportion of total charges. While inpatient visits made up only 3.0% of all visits, 68.7% of total charges were inpatient-related (Table 3). ED visits accounted for 16.1% of all TBI-related charges.

**Table 3:** **Total charges of pediatric traumatic brain injury-related visits at one midwest children’s hospital from August 1, 2010 through July 31, 2011 by diagnosis and by clinical site/setting**

	Total # visits n (%)	Total charges total $ (%)
**Diagnosis**		
Head injury, unspecified	2,574 (47.4%)	$1,649,203 (12.6%)
Concussion	2,204 (40.6%)	$2,498,255 (19.0%)
Brain injury with contusion, laceration, or hemorrhage	388 (7.1%)	$3,556,358 (27.1%)
Fracture of the vault or base of the skull	186 (3.4%)	$4,870,781 (37.1%)
Other and unqualified multiple fractures of the skull	77 (1.4%)	$556,949 (4.2%)
**Total**	5,429 (100.0%)*	**$13,131,547 (100.0%)**
**TBI severity**		
Mild TBI**	4,992 (92.0%)	$5,869,449 (44.7%)
Other TBI	437 (8.0%)	$7,262,098 (55.3%)
**Total**	5,429 (100.0%)*	**$13,131,547 (100.0%)**
**Clinical site/setting**		
Emergency	1,776 (32.7%)	$2,108,271 (16.1%)
Clinic outpatient	1,704 (31.4%)	$649,523 (4.9%)
Urgent care	1,498 (27.6%)	$397,603 (3.0%)
Diagnostic/Therapy outpatient	242 (4.5%)	$306,402 (2.3%)
Inpatient	163 (3.0%)	$9,015,770 (68.7%)
Observation	42 (0.8%)	$392,259 (3.0%)
Organ procurement	4 (0.1%)	$261,720 (2.0%)
**Total**	5,429 (100.0%)*	**$13,131,547 (100.0%)**

*Totals do not sum to 100.0% due to rounding.

**The mild TBI (mTBI) category was constructed from various codes from across the five diagnosis categories.

**Figure 1:**
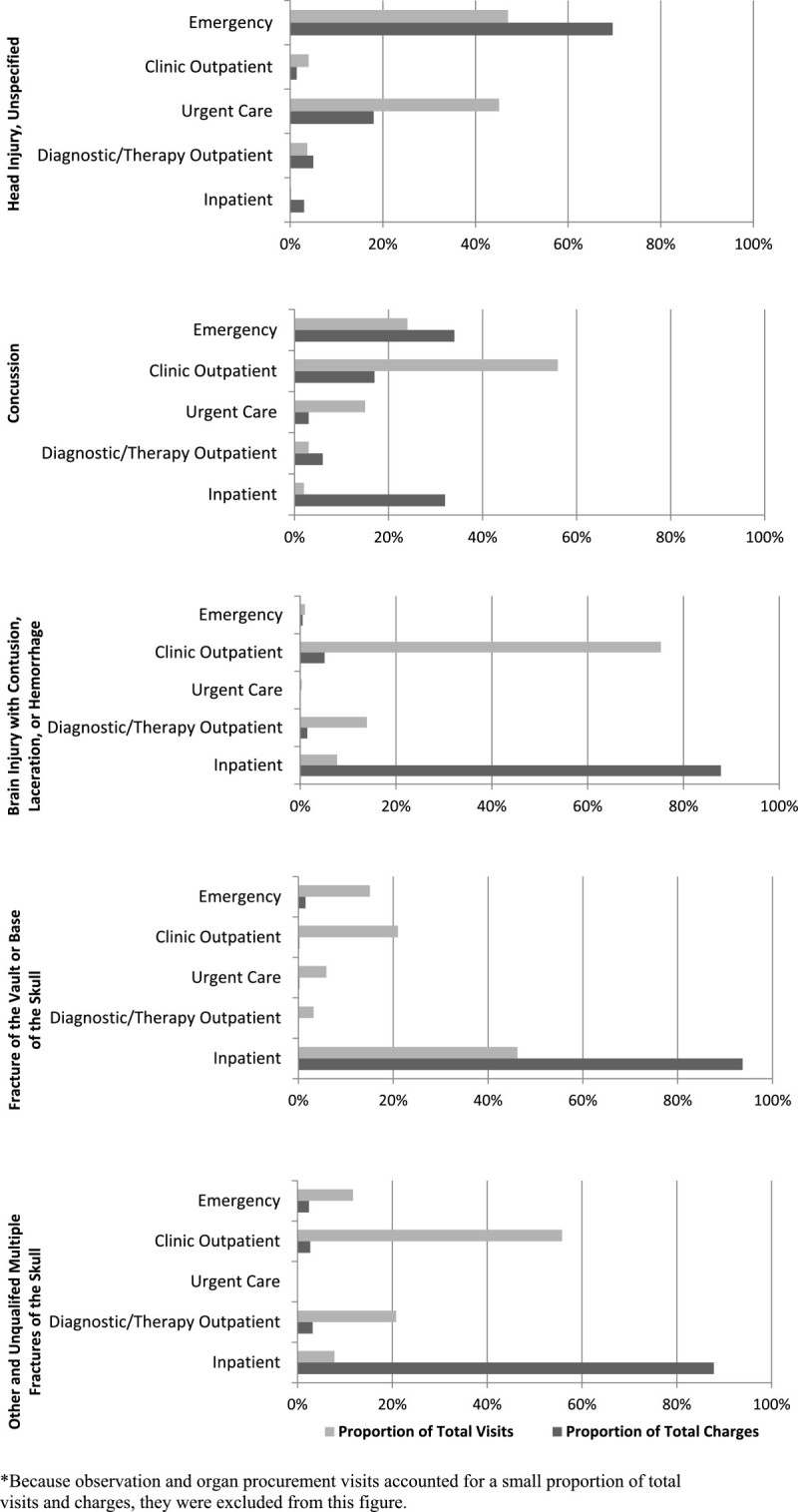
Total charges of pediatric traumatic brain injury-related visits at one midwest children’s hospital from August 1, 2010 through July 31, 2011 by diagnosis* and clinical site/setting.

The vast majority of total charges for brain injury with contusion, laceration, or hemorrhage (87.8%), fracture of the vault or base of the skull (93.7%), and other unqualified multiple fractures of the skull (87.8%) visits, which are typically more severe TBIs, were inpatient-related (Figure 1). Among all concussion-related visits, the highest proportions of total charges were inpatient (31.5%) and ED-related (34.4%); however, a substantial proportion of total charges were associated with clinic outpatient visits (16.9%). Among unspecified head injury-related visits (ICD-9-CM 959.01), which were all classified as mTBI-related, 18.4% of medical charges were from urgent care visits.

Although mTBIs accounted for 92.0% of all TBI-related visits, they accounted for 44.7% of all TBI-related charges (Table 3). The highest proportions of mTBI-related total charges were emergency (34.8%), urgent care (29.7%), and clinic outpatient-related (29.1%). mTBIs treated in the ED had a higher median total charge per visit (median: $356) than mTBIs treated in the urgent care (median: $167) (p < 0.001) or clinic outpatient setting (median: $274) (p < 0.001).

#### Individuals with one visit

The total charge for individuals with one visit was $5,836,662 (44.4% of total charges), and the median total charge per patient was $320. There were a similar proportion of total charges related to unspecified head injury (23.6%), concussion (23.2%), brain injury with contusion, laceration, or hemorrhage (23.1%), and fracture of the vault or base of the skull (29.5%) (Table 4). While inpatient visits made up only 2.7% of total visits among individuals with one visit, they accounted for 55.0% of all charges (Table 4). ED visits, which made of 6.0% of all visits among this group, accounted for 28.0% of total charges.

**Table 4:** **Total charges of pediatric traumatic brain injury-related visits at one midwest children’s hospital from August 1, 2010 through July 31, 2011 by number of visits per individual**

	Total charges total $ (%)	
	Individuals with one visit (n = 3,185)	Individuals with two or more related visits (n = 740)*
**Diagnosis**		
Head injury, unspecified	$1,378,301 (23.6%)	$232,246 (3.2%)
Concussion	$1,355,685 (23.2%)	$1,122,136 (15.5%)
Brain injury with contusion, laceration, or hemorrhage	$1,348,338 (23.1%)	$2,194,827 (30.4%)
Fracture of the vault or base of the skull	$1,719,853 (29.5%)	$3,150,929 (43.6%)
Other and unqualified multiple fractures of the skull	$34,486 (0.6%)	$519,808 (7.2%)
**Total**	**$5,836,662 (100.0%)**	**$7,219,945 (100.0%)**
**TBI severity**		
Mild TBI**	$3,231,763 (55.4%)	$2,562,747 (35.5%)
Other TBI	$2,604,899 (44.6%)	$4,657,199 (64.5%)
**Total**	**$5,836,662 (100.0%)**	**$7,219,945 (100.0%)**
**Clinical Site/Setting**		
Emergency	$1,636,051 (28.0%)	$436,763 (6.0%)
Clinic outpatient	$96,965 (1.7%)	$531,581 (7.4%)
Urgent care	$329,491 (5.6%)	$59,301 (0.8%)
Diagnostic/Therapy outpatient	$104,942 (1.8%)	$191,765 (2.7%)
Inpatient	$3,209,383 (55.0%)	$5,806,386 (80.4%)
Observation	$198,110 (3.4%)	$194,149 (2.7%)
Organ procurement	$261,720 (4.5%)	$0 (0.0%)
**Total**	**$5,836,662 (100.0%)**	**$7,219,945 (100.0%)**

*Individuals with two or more unrelated visits (n = 46) are excluded from this table (total charges = $74,939).

**The mild TBI (mTBI) category was constructed from various codes from across the five diagnosis categories.

#### Individuals with two or more related visits

The total charge for patients with two or more related visits was $7,219,945 (55.0% of total charges), and the median total charge per patient was $1,254. The majority of charges were associated with fracture of the vault or base of the skull (43.6%) and brain injury with contusion, laceration, or hemorrhage (30.4%) (Table 4). While inpatient visits made up only 3.6% of total visits in this group, they accounted for 80.4% of all charges. Approximately 10% of all charges among patients with two or more related visits were from clinic outpatient visits (7.4%) and diagnostic/therapy outpatient visits (2.7%).

## Discussion

This study was the first to provide a more comprehensive estimate of the direct medical charges associated with pediatric traumatic brain injuries by analyzing total medical charges across multiple clinical settings at one large US Midwest Children’s Hospital. Not surprisingly, inpatient and ED visits accounted for the highest proportion of pediatric TBI-related charges (68.7% and 16.1%, respectively); however, more than 15% of all charges were associated with visits to other clinical settings including clinic outpatient, urgent care, and diagnostic/therapy outpatient. This study demonstrates that previous studies which used ED and inpatient-based statistics alone to estimate the medical charges of pediatric TBI have undoubtedly underestimated the true medical charges associated with this serious public health issue.

As expected, children with potentially “more severe” TBIs, such as brain injury with contusion, laceration, or hemorrhage or fracture of the vault or base of the skull, were more likely to require inpatient stays and have two or more visits. As a result, these patients also had higher total medical charges. However, we also found that approximately 10% of all charges among patients with two or more related visits were from clinic outpatient visits (7.4%) and diagnostic/therapy outpatient visits (2.7%). This demonstrates that the bulk of the charges associated with more severe TBIs stems from the initial visit and that subsequent visits for follow up care are relatively inexpensive. Future research should determine if increasing the number of follow up visits might improve long term outcome for more severe TBI given the relatively low cost of follow up care compared to the severity of this injury and the potential for long term negative health consequences. Even among patients with more severe TBIs, failing to consider the medical charges of all visits across all clinical settings will underestimate the true cost of this important injury.

There were differences in charges by diagnosis and clinical site/setting, especially among pediatric mTBIs compared to “more severe” TBIs. Previous studies have found that mTBI-related visits may account for up to 70% to 90% of all TBIs, (Cassidy et al. 2004; Faul et al. 2010) and, in the current study, we found mTBI represented 92.0% of all TBI visits. Yet mTBIs accounted for 44.7% of all TBI-related charges. Among concussion-related visits, the highest proportions of total charges were inpatient (31.5%) and ED-related (34.4%); however, a substantial proportion of total charges were associated with clinic outpatient visits (16.9%). Similarly, 18.4% of unspecified head injury-related charges were from urgent care visits. Given the high incidence of mTBIs and the finding that mTBIs often present to clinical settings other than the ED or inpatient coupled with the substantial charges associated with mTBIs, it is important to try capture as many of these injuries as possible when evaluating the true clinical, economic, and public health burden of pediatric TBIs.

We also found that mTBIs treated in the ED had a median total charge per visit that was more than two times the median total charge per visit for mTBIs treated in urgent care settings. Additional educational efforts are needed to help the public understand the signs and symptoms of concussion and the importance of seeking appropriate medical advice and proper management to avoid unnecessary and costly visits to emergency departments. However, because of the current debate surrounding the diagnostic criteria for concussion, it is difficult to determine if injuries presenting to various health care settings are truly dissimilar, or if these differences result from varying diagnostic criteria used by physicians practicing in different clinical settings. More research is needed to examine how ICD-9-CM codes are being used in each type of clinical setting, particularly ICD-9-CM code 959.01 (head injury, unspecified) and other codes used for mTBI, if these codes are being used correctly, (Bazarian et al. 2006; Coronado et al. 2012b; Powell et al. 2008) and how potential clinical setting differences may affect estimates of the incidence and associated charges of TBI.

In a previous study using the same data, we found differences in patterns of pediatric TBIs by patient demographics (Collins et al: Clinical Presentation Patterns and Settings for Pediatric Traumatic Brain Injury, Currently under review at J Head Trauma Rehabil). More specifically, type of insurance, diagnosis, and number of visits varied by race. A higher proportion of white patients were diagnosed with a concussion, had commercial insurance, and had two or more related visits while a higher proportion of black/African American patients were diagnosed with an unspecified head injury, had Medicaid, and had only one visit. Because these factors were so strongly associated, we hypothesized these differences were more likely related to socioeconomic status (SES) and/or primary payor than patient’s race. We found significant differences in median total charge per patient by gender, race, and primary payor. These findings suggest that children with higher SES may be receiving different care for TBIs than children with lower SES. Additional research is needed to more clearly understand how TBI visits differ by patient characteristics such as SES, if these characteristics influence medical decisions including when and where to seek care, and how patient characteristics and treatment decisions affect charges.

This study had several limitations. First, generalizability may be limited as data were restricted to one, large Midwest Children’s Hospital. Medical charges may vary by institution or location in the US. However, multiple clinical settings in the hospital network were included, this Children’s Hospital has a large geographic catchment area, and limiting this study to a single institution provided a consistency of clinical records. Second, as in all studies, we had to establish a finite study period, and we may have missed the initial injury visit for some TBI-related visits that occurred at the beginning of the study period and/or missed follow up visits that occurred after the end of the study period. However, as a relatively large number of patients who had two or more visits related to one injury event were included in the study, we are confident that those described here are representative. In addition, an academic year time period was used in an effort to capture sports-related initial injury visits in our study period. Third, it was not possible to determine if all medical charges were directly related to the principal diagnosis of TBI or potentially associated with concurrent injuries and/or comorbidities. Fourth, we were unable to obtain information regarding the cause of pediatric TBIs which might influence cost. Fifth, this study used total medical charges, which included all direct charges such as physician charges, pharmaceutical charges, laboratory charges, facility charges, etc. It is important to note that total medical charges may vary greatly from actual medical costs. Future studies are needed to determine how the medical cost of pediatric TBI varies across various clinical settings as well as across multiple hospitals in different regions of the country. Finally, like previous studies, the present study may have actually underestimated the true medical charges of pediatric TBIs as TBIs treated at locations outside of the hospital network would have been missed. Despite these limitations, this study is the first to capture direct medical charges of pediatric traumatic brain injuries across all health care settings associated with a large US Children’s Hospital. The findings presented here should improve future knowledge of the medical cost of pediatric TBI by driving researchers to conduct future studies in samples including multiple clinical settings.

## Conclusions

Pediatric TBIs present to a wide variety of clinical settings, and differences exist in total charges by diagnosis, severity of the injury, and clinical site/setting. These findings clearly demonstrate that previous research utilizing only inpatient or ED data has underestimated the charges associated with pediatric TBIs. Researchers investigating TBIs charges must be encouraged to capture data across the full spectrum of clinical care settings in order to provide both clinicians and policy makers with more complete and current information. Only when such data from multiple clinical settings are presented from large national samples will we be able to evaluate the true medical cost of pediatric TBI.

## Authors’ original submitted files for images

Below are the links to the authors’ original submitted files for images. Authors’ original file for figure 1
