# Supplementary material for: Direct medical charges of pediatric traumatic brain injury in multiple clinical settings
Source: Inj Epidemiol. 2014 May 6;1(1):13. doi: 10.1186/2197-1714-1-13 (PMC5005782; doi:10.1186/2197-1714-1-13)
Supplement: Supplementary file 1 — Authors’ original file for figure 1 [file 40621_2013_13_MOESM1_ESM.pdf]

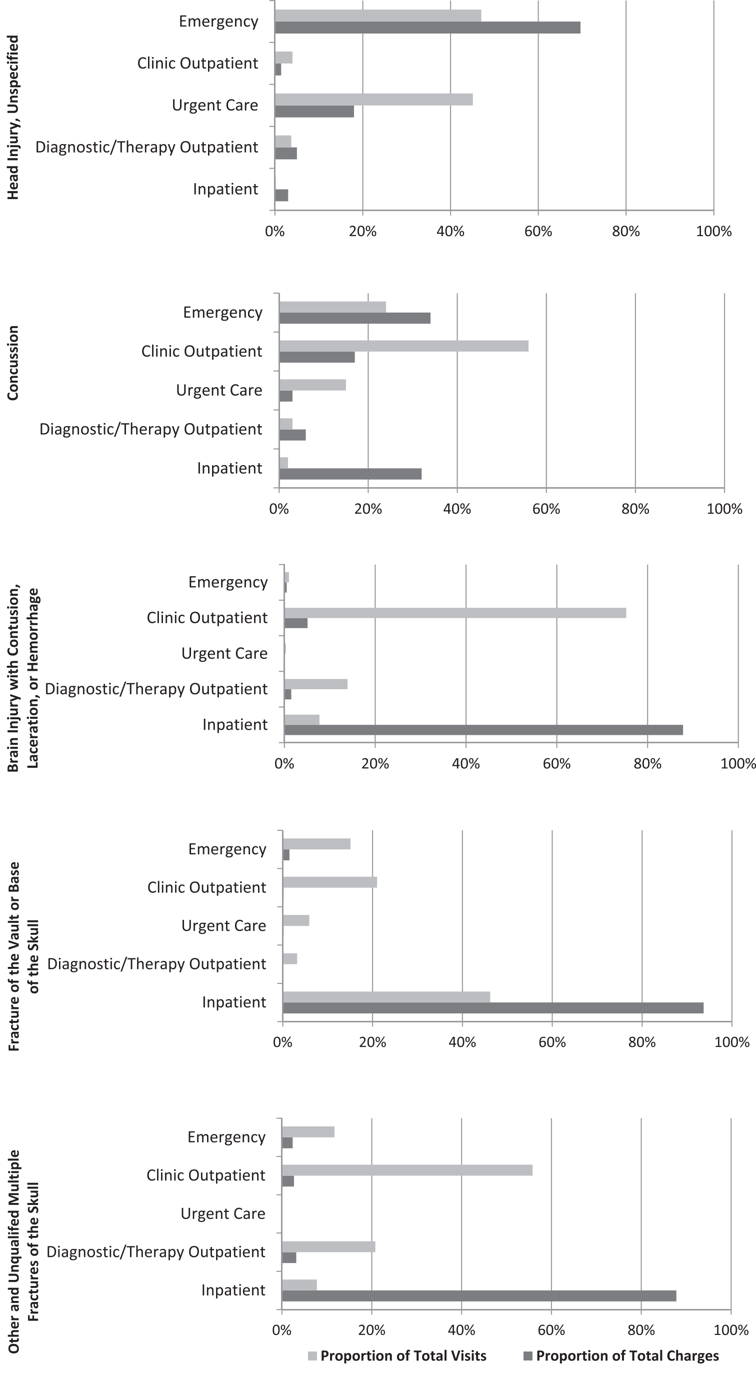

*Because observation and organ procurement visits accounted for a small proportion of total visits and charges, they were excluded from this figure.
